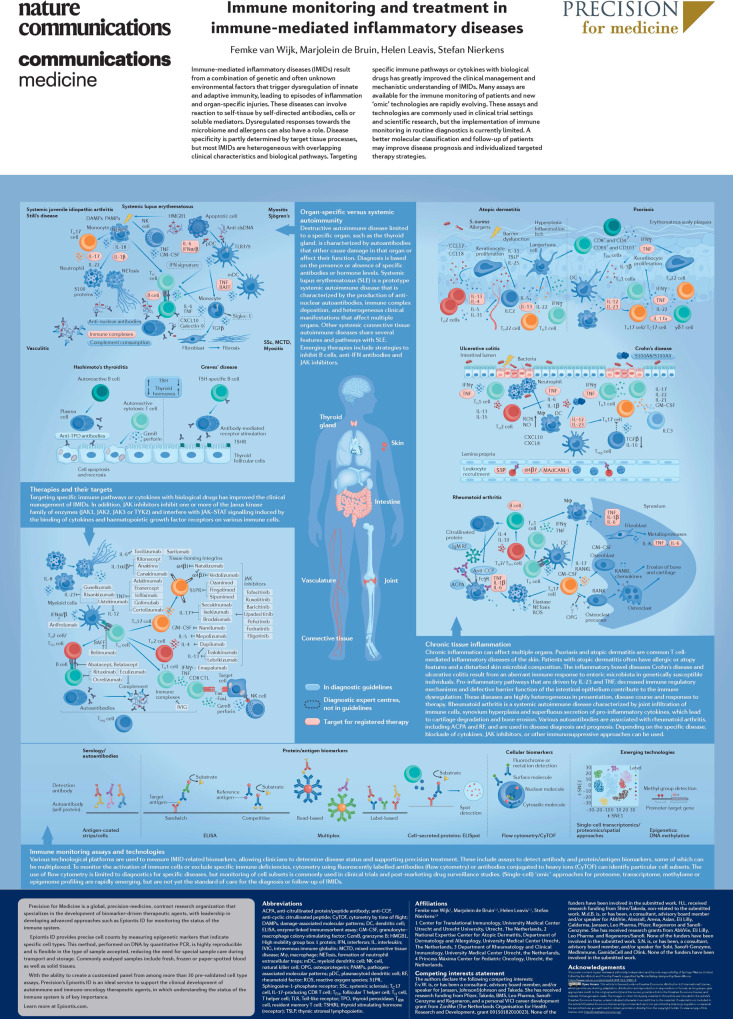# Immune monitoring and treatment in immune-mediated inflammatory diseases

**DOI:** 10.1038/s41467-022-30891-7

**Published:** 2022-06-07

**Authors:** Femke van Wijk, Marjolein de Bruin, Helen Leavis, Stefan Nierkens

**Affiliations:** 1 Center for Translational Immunology, University Medical Center Utrecht and Utrecht University, Utrecht, The Netherlands; 2 National Expertise Center for Atopic Dermatitis, Department of Dermatology and Allergology, University Medical Center Utrecht, Utrecht, The Netherlands; 3 Department of Rheumatology and Clinical Immunology, University Medical Center Utrecht, Utrecht, The Netherlands; 4 Princess Máxima Center for Pediatric Oncology, Utrecht, The Netherlands

**Keywords:** Biomarkers, Immunopathogenesis, Rheumatic diseases, Gastrointestinal diseases, Immunological disorders, Immunological techniques

## Abstract

Immune monitoring assists in the diagnosis and clinical management of immune-mediated inflammatory diseases.

Immune-mediated inflammatory diseases (IMIDs) can occur in a number of organ systems as a result of aberrant innate and adaptive immune responses to genetic and environmental triggers. Immune-monitoring technologies are used to detect disease-relevant immune biomarkers, either to enable diagnosis or monitor response to therapy. Immune monitoring is predominantly used in the research setting at present but routine clinical use is increasing. This poster explores the mechanisms underlying a number of common IMIDs, biomarkers relevant to the diagnosis or monitoring of IMIDs, and therapies used to treat these diseases.

This poster is freely available online thanks to support from Precision for Medicine.

The poster has been peer reviewed and, as always, Springer Nature retains sole responsibility for all editorial content.